# When proteases reshape barriers: Basement membrane remodelling in development, wound healing and tumour progression

**DOI:** 10.1111/febs.70585

**Published:** 2026-05-19

**Authors:** Clara Legendre, Guila Dayan, Patricia Rousselle

**Affiliations:** ^1^ Laboratoire de Biologie Tissulaire et Ingénierie Thérapeutique, UMR 5305, CNRS, Univ. Lyon 1, SFR BioSciences Gerland‐Lyon Sud France

**Keywords:** basement membrane, cancer invasion, extracellular matrix, invadopodia, keratinocyte, matrix metalloproteinases, morphogenesis, podosome, wound healing

## Abstract

Basement membranes (BMs) are dynamic extracellular matrices whose remodelling controls tissue morphogenesis, regeneration and malignant progression. This review examines how a shared protease‐ and extracellular matrix (ECM)‐based machinery reshapes laminin‐ and collagen IV‐rich BMs during development and wound repair, and how the altered levels, localisation and regulation of these components drive cancer invasion. In development, patterned BM thinning, perforation and stiffening driven by MMPs, MT‐MMPs, ADAMTS proteases and nonproteolytic regulators generate gradients that guide branching, tissue elongation and axis formation. In cutaneous wound healing, many of the same proteases (including MMP‐1, MMP‐9, MT1‐MMP, stromelysins, serine proteases and cathepsins) act in transient, spatially restricted patterns so that keratinocytes remodel a provisional matrix at the wound edge and then rebuild a mature dermal–epidermal BM. By contrast, in carcinomas, invadopodia enriched in MT1‐MMP, MMP‐2, MMP‐9 and related proteases concentrate pericellular proteolysis to perforate BMs, while collagen IV ‘escape tracks’ and laminin‐derived cryptic fragments promote proliferation, survival, epithelial–mesenchymal transition and angiogenesis. Finally, we highlight emerging therapeutic strategies, including isoform‐ and site‐selective protease inhibitors and protease‐responsive or protease‐modulating biomaterials, that seek to locally rebalance this protease‐ECM programme to restore BM integrity in chronic wounds and limit invasive growth in cancer.

## Graphical abstract

Basement membrane (BM) homeostasis relies on a balance between integrity, controlled remodelling and pathological degradation. Increased protease expression beneath the BM, along with reduced levels of endogenous protease inhibitors, drives the transition from a continuous, protective barrier to a discontinuous interface. This facilitates protease‐dependent tissue remodelling during normal physiological processes and, when uncontrolled, permits cell invasion, disrupts basement membrane architecture and contributes to disease‐associated matrix instability in various pathological contexts.

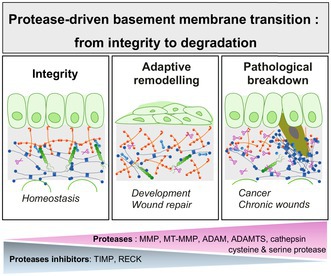

**Abbreviations**
ADAM: a disintegrin and metalloproteinase
ADAMTS: a disintegrin and metalloproteinase with thrombospondin motifs
BM: basement membrane
ECM: extracellular matrix
LM: laminin
MMP: matrix metalloproteinase
MT‐MMP: membrane‐type matrix metalloproteinase
TIMP: tissue inhibitor of metalloproteinases

## Introduction

**Fig. 1:**
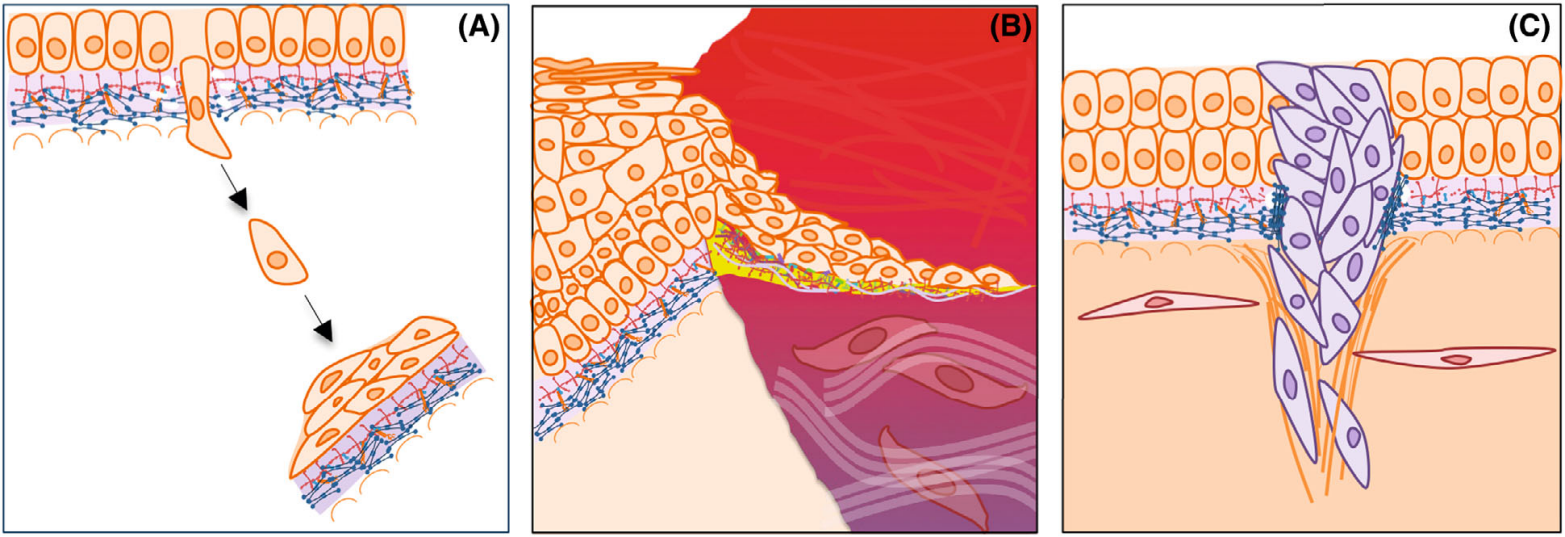
Context‐dependent basement membrane remodelling in development, wound healing and cancer. (A) During development, basement membrane (BM) remodelling is spatially patterned, with transient, localised perforations allowing controlled cell infiltration that drives morphogenesis. (B) In acute skin wounds, the dermal–epidermal BM undergoes local degradation to permit keratinocyte migration over a provisional laminin‐rich matrix, followed by reassembly of a mature, collagen IV–stabilised BM that restores barrier function. (C) In epithelial cancers, similar remodelling mechanisms become chronic and misregulated, leading to persistent BM breaching and invasion of tumour cells into the underlying stroma.

Basement membranes (BM) are thin, highly specialised extracellular matrix (ECM) networks located at the basal side of polarised epithelia, endothelia, muscle, fat and nerve cells [1]. Evolutionarily conserved from invertebrates to mammals, BMs surround organs and tissues, providing structural support and defining tissue boundaries [2]. These matrices act as scaffolds for cell adhesion and influence cellular signalling pathways and behaviours, such as migration, proliferation and differentiation [3, 4]. Consequently, BMs play essential roles in morphogenesis, growth‐factor signalling and the establishment of cell polarity [5]. The primary structural framework of BMs consists of two interconnected polymeric networks, laminin (LM) and type IV collagen, bridged mainly by nidogen and the heparan sulphate proteoglycan perlecan [3, 6], and embedded within a highly ordered assembly of additional components (Fig. 1). These include heparan sulphate proteoglycans (e.g. agrin, collagens XV and XVIII), as well as fibulins, small leucine‐rich proteoglycans and other matricellular glycoproteins, which modulate BM architecture, growth‐factor sequestration and cell–matrix interactions in a tissue‐ and context‐dependent manner [7, 8]. Biochemical and genetic studies in humans and model organisms have produced a canonical model of BM assembly [1, 9, 10, 11]. Both collagen IV and LM are trimeric proteins that self‐assemble into distinct interconnected networks. LM is considered the initiator of BM assembly and self‐assembles at the cell surface, where it nucleates BM formation [1, 3, 12], whereas collagen IV confers mechanical strength through covalent cross‐linking that provides tensile integrity [13]. All LMs consist of three different gene products, α, β and γ chains assembled into a cross‐shaped αβγ heterotrimer. These chains oligomerise within the endoplasmic reticulum via their C‐terminal domains, forming a triple‐stranded α‐helical coiled‐coil stabilised by disulphide bonds at both ends [14, 15]. The five α (α1–α5), four β (β1–β4) and three γ (γ1–γ3) chains identified by cDNA sequencing can combine to produce at least 18 heterotrimeric isoforms (Table 1) [16]. Some LM genes generate multiple isoforms through alternative splicing, producing short‐chain and long‐chain variants [17].

**Table 1:** Laminin isoforms.

Name	Chain assembly	Previous name	Original name
Laminin‐111	α1β1γ1	1	EHS laminin
Laminin‐211	α2β1γ1	2	Merosin
Laminin‐121	α1β2γ1	3	S‐laminin
Laminin‐221	α2β2γ1	4	S‐merosin
Laminin‐3A32	α3Aβ3γ2	5 or 5A	Kalinin, epilegrin, nicein, ladsin
Laminin‐3B32	α3Bβ3γ2	5B	
Laminin‐3A11	α3Aβ1γ1	6 or 6A	K‐laminin
Laminin‐3A21	α3Aβ2γ1	7 or 7A	KS‐laminin
Laminin‐411	α4β1γ1	8	
Laminin‐421	α4β2γ1	9	
Laminin‐511	α5β1γ1	10	Drosophila‐like laminin
Laminin‐521	α5β2γ1	11	
Laminin‐213	α2β1γ3	12	
Laminin‐423	α4β2γ3	14	
Laminin‐523	α5β2γ3	15	
Laminin‐522^a^	α5β2γ2		
Laminin‐212^b^	α2β1γ2		
Laminin‐222^b^	α2β2γ2		
Laminin‐333^c^	α3β3γ3		

^a^ Laminin‐522 reported so far solely in bone marrow.

^b^ The existence of either LM‐212 or LM‐222 is proposed based on studies of peripheral nerves in wild‐type and LM α2 chain‐deficient mice.

^c^ Laminin‐333 is expressed at the apical specialisation of adult rat testes, rather than in the testicular basement membrane.

Each BM contains at least one LM isoform, and some contain two or three, with their expression and assembly tightly regulated in space and time to support tissue‐specific and developmental functions [18]. Certain isoforms, particularly those containing α1 or α5, are expressed early in embryogenesis and serve as developmental markers, whereas others appear later in more restricted tissue contexts. In general, α5 exhibits the broadest distribution, α1 is the most restricted, α2 predominates in mesoderm‐derived tissues, such as cardiac and skeletal muscle, α4 is enriched in endothelial and mesenchymal compartments, and α3, β3 and γ2 are largely confined to epithelial BMs [19]. The β1 and β2 chains typically display mutually exclusive expression, while γ1 is present in all BMs [20]. LMs are essential during embryogenesis, and loss‐of‐function mutations in key LM genes frequently cause mid‐gestation lethality, limiting analysis of their roles at later stages [21]. In epithelial tissues, the BM is additionally mechanically coupled to basal cells through hemidesmosomes, multiprotein adhesion complexes built around integrin α6β4 and collagen XVII that bind LM‐332, thereby linking the intermediate filament cytoskeleton to the BM and ensuring stable epithelial–stromal adhesion [22, 23].

Collagen IV also exists as multiple isoforms determined by the combination of six cloned and sequenced α(IV) chains, which show tissue‐specific expression [24]. The α1 (IV) and α2 (IV) chains are present in all BMs that form extensively cross‐linked networks providing fundamental mechanical support and load‐bearing capacity to the BM [10, 24, 25].

## Basement membrane remodelling in development, repair and cancer

Heterogeneity in BM composition, turnover and mechanical stiffness is crucial for organ development and morphology, and its dysregulation is linked to diverse diseases [26, 27]. Dynamic BM remodelling underscores its importance in physiological processes, such as embryonic morphogenesis, as well as in pathological contexts including cancer invasion [28, 29, 30]. During development in worms, flies and mammals, coordinated changes in BM composition, thickness and stiffness, together with localised proteolysis, enable epithelial bending, branching and tissue elongation that sculpt organ shape and positioning [31]. At regulated invasion sites, such as the *C. elegans* anchor cell, invasive cells in *Drosophila* discs, and the primitive streak in mammals, focal BM effacement or perforation permits cells to cross barriers [32].

Aberrations in BM components during development frequently cause severe, often perinatal diseases and mutations in LMs underlie disorders affecting skin, muscle and nerves [33, 34]. In adult tissues such as skin, similar remodelling principles govern wound repair: transient BM degradation followed by LM‐ and collagen IV‐enriched reassembly enables keratinocyte migration, re‐epithelialisation and restoration of barrier function [35, 36, 37, 38].

By contrast, in epithelial cancers, these developmental and wound‐associated programmes are chronically subverted. BM components are aberrantly expressed and proteolytically processed, and the LM‐collagen IV network is persistently breached at invasive fronts where tumour cells cross the epithelial–stromal boundary [39, 40, 41, 42, 43]. These converging observations support the view that localised, often protease‐dependent BM breaching is a key event enabling cellular transmigration and tissue remodelling. Together, the dynamic composition and organisation of BMs maintain tissue architecture and regulate cell fate decisions throughout development, homeostasis and disease. A common repertoire of BM remodelling mechanisms underpins tissue morphogenesis (Fig. 1A), regeneration (Fig. 1B) and malignant progression (Fig. 1C), with biological context and resolution determining whether the outcome is organ formation, repair or cancer.

## Zinc metalloproteinases and their inhibitors in basement membrane remodelling

Matrix metalloproteinases (MMPs) are a family of secreted and membrane‐bound, zinc‐dependent endopeptidases capable of degrading most ECM components, including BM, and play key roles in morphogenesis, tissue repair and the progression of inflammatory and neoplastic diseases [44, 45, 46]. The vertebrate MMP family comprises 25 related gene products (24 in mammals), classically grouped as collagenases, gelatinases, stromelysins, matrilysins, membrane‐type MMPs (MT‐MMPs) and other MMPs, whose expression and activation are tightly regulated by cytokines, growth factors and ECM signals [45, 47]. Throughout development, wound healing and cancer, the impact of MMPs on BM integrity is determined not only by their expression and activation, but also by inhibition through tissue inhibitors of metalloproteinases (TIMP‐1 to TIMP‐4) and related regulators such as RECK, a GPI‐anchored glycoprotein that suppresses the activity and cell‐surface behaviour of MMP‐2, MMP‐9 and MT1‐MMP, thereby helping to preserve BM integrity in development and disease [48, 49]. TIMP‐1 to TIMP‐4 bind active MMPs in a 1 : 1 stoichiometry and can also regulate the activation of pro‐MMPs at the cell surface, exerting tight spatiotemporal control over pericellular proteolysis [50]. Under physiological conditions, a balanced MMP‐TIMP system supports controlled BM remodelling during morphogenesis and acute repair, whereas shifts in this equilibrium, such as increased MMP activity and/or reduced TIMP expression, are hallmarks of chronic wounds, fibrotic disease and many cancers, where excessive or mislocalised BM degradation promotes invasion, angiogenesis and tissue destabilisation. Rather than absolute MMP levels, it is this spatiotemporal balance between MMPs and TIMPs that ultimately determines whether BM remodelling is transient and permissive or excessive and destructive in a given context [51, 52]. A Disintegrin And Metalloproteinases (ADAMs) are membrane‐anchored zinc proteases that cleave cell‐surface and pericellular substrates, including adhesion receptors and growth‐factor precursors, thereby indirectly influencing BM remodelling through effects on cell‐matrix adhesion and signalling [53, 54]. A Disintegrin And Metalloproteinase with Thrombospondin motifs (ADAMTS) proteases are secreted, ECM‐associated metalloproteinases that process proteoglycans and selected BM‐adjacent components and can regulate BM heterogeneity, angiogenesis and tumour‐stroma interactions in development, wound healing and cancer [4, 55, 56]. As with MMPs, their impact on BM integrity does not simply reflect their presence or absence but depends on when and where individual ADAM and ADAMTS family members are deployed, and on how their activities are integrated with MMP‐TIMP networks in a given tissue context.

During development and acute wound healing, this multi‐layered regulation generates tightly patterned, short‐lived bursts of MMP and MT‐MMP activity at defined cell‐BM interfaces, such as branching tips or the wound edge, permitting focal BM perforation and reassembly while preserving overall barrier integrity. By contrast, in chronic wounds and tumours, persistent inflammatory signalling, altered integrin and growth‐factor pathways, and changes in TIMP and RECK expression disturb this balance, favouring sustained, mislocalised proteolysis, chronic BM weakening and progressive tissue destabilisation. Other extracellular zinc metalloproteinases, such as BMP1/tolloid‐like proteinases involved in BM assembly and wound repair, also contribute to BM remodelling but are not discussed here [57, 58].

## Protease‐guided basement membrane remodelling in development and morphogenesis

Proteolytic processing of LMs in development can weaken and re‐pattern BM barriers, as well as generate bioactive fragments with specific signalling roles. In the testis, MMP‐2 cleavage of the LM γ3 chain within LM‐333, localised at the apical ectoplasmic specialisation of Sertoli cells, yields a 50‐amino‐acid peptide that modulates tight junction permeability and spermatogenic function [59, 60, 61, 62, 63]. An 80‐kDa LM α2 C‐terminal fragment, possibly generated by MMP‐9, similarly contributes to blood–testis barrier remodelling and spermatogenesis [64]. In embryonic stem cells, MMP‐2 cleavage of the short arm of LM β1 is predicted to disassemble the LM network and release a β1 fragment that promotes α3β1‐dependent adhesion, linking LM proteolysis to lineage‐specific adhesion and signalling [65]. During zebrafish skeletal muscle development, LM also mediates MMP‐11 activity that regulates fibronectin levels at the myotendinous junction, coordinating ECM composition with muscle attachment and growth [66].

Beyond simple degradation by proteases, BM collagen IV networks become spatially heterogeneous as organs grow and change shape. In the Drosophila egg chamber, collagen IV density is reduced at the posterior pole and the micropattern of aligned fibre‐like structures is altered; ADAMTS proteases act as key regulators of this heterogeneity, which is required for proper organ shape [55]. BM dynamics reflect not only local proteolysis and deposition but also regulated trafficking and long‐range delivery of BM components from distant tissues [67]. In the egg chamber, follicle morphogenesis depends on an anterior–posterior gradient of collagen IV that sets tissue mechanical properties and elongation; this gradient arises through post‐transcriptional control by the metalloprotease ADAMTS‐A in the secretory pathway, which limits collagen IV deposition and thereby patterns the BM [67]. Consistent with this view, endogenous tagging of 29 BM components and receptors with mNeonGreen in *C. elegans* showed that different tissues assemble BMs with distinct compositions and turnover rates, highlighting BM remodelling and patterning as widespread developmental features [68].

At the tissue scale, work in Drosophila egg chambers and mammalian glands indicates that epithelia can use protease‐dependent BM orientation, translocation and local accumulation to shape organ architecture. Proteolytic remodelling is required for branching morphogenesis in pulmonary, salivary and mammary glands, where MMPs permit epithelial outgrowth and liberate growth factors that drive branching [69, 70, 71, 72]. During chick development, before gastrulation and epithelial–mesenchymal transition, the BM moves with epiblast cells during primitive streak formation, suggesting that cells carry or accumulate BM as they migrate [73]. In *C. elegans* uterine–vulval morphogenesis, BM disruption is initiated by anchoring‐cell invadopodia and the initial perforation is subsequently widened by mechanical displacement and sliding of the BM [74, 75].

Live imaging of branching organs shows that the BM surrounding developing epithelia is highly dynamic, undergoing local perforation and global remodelling via MMP activity and actomyosin contractility while maintaining an overall intact epithelial compartment. Pharmacological inhibition of MMPs impairs BM perforation in mouse salivary glands, underscoring the requirement for proteolytic remodelling in branching [76]. Similar MMP‐dependent BM perforation and remodelling accompany disc eversion during Drosophila metamorphosis, echoing mechanisms used in tumour invasion [31].

Recent work in the mouse embryo indicates that protease‐dependent BM perforations can act as instructive cues rather than passive consequences of invasion. In early mouse embryos, MMP‐mediated, spatiotemporally regulated BM perforation after implantation is essential for coordinated growth, morphogenesis and gastrulation [77]. Asymmetric, protease‐dependent perforations between the visceral endoderm and epiblast arise before distal visceral endoderm migration and are required to guide its collective movement, which breaks symmetry and establishes the anterior–posterior axis [78]. Similar anterior‐biased perforation patterns in human embryos and stem cell models suggest that patterned BM remodelling is a conserved mechanism for directing early mammalian morphogenesis [79].

Comparative studies also highlight that normal and pathological cells exploit distinct protease‐dependent and protease‐independent strategies to traverse native BMs. For example, macrophages and cancer cells both deploy membrane‐anchored MMPs to access and remodel BMs, but only macrophages can switch to an actomyosin‐dependent, protease‐independent invasion mode by deforming through pre‐existing pores that carcinoma cells cannot use [40]. Leukocytes provide another example of physiological low‐damage BM traversal: they cross vessel and lymphatic BMs through pre‐existing pores that enlarge mechanically as the cell passes, with little requirement for extensive proteolysis [80].

BMs are remodelled not only by proteases but also by nonproteolytic regulators that adjust network organisation, pore size and stiffness. Netrin‐4 is a LM‐related BM component that binds LM γ1 with high affinity and, at sufficient concentrations, interferes with LM polymerisation, enlarges BM pores and softens LM networks without requiring protease activity [79, 81, 82, 83, 84, 85]. This rheological modulation supports a ‘netrin‐4 : LM rheostat’ model in which netrin‐4 levels tune BM stiffness and permeability to control branching morphogenesis, vascular maturation and epithelial fusion [79, 86, 87]. Additional nonproteolytic inputs, including flow‐induced remodelling of endothelial BMs, LM polarisation and LOXL2‐mediated collagen IV cross‐linking, further shape BM mechanics and topology during development [88, 89, 90].

These examples show how tightly patterned, transient, spatially restricted MMP and ADAMTS activity permit BM perforation, sliding and reassembly during morphogenesis, in marked contrast to the sustained, mislocalised proteolysis that underlies chronic BM weakening and invasive tumour growth.

## Protease‐guided basement membrane remodelling in cutaneous wound healing

Cutaneous wound healing relies on a transient, spatially restricted protease‐dependent breakdown and reassembly of the dermal–epidermal BM [38]. At the wound edge, the pre‐existing BM is locally degraded and replaced by a provisional matrix rich in fibrin, fibronectin, LMs and tenascin C, which supports keratinocyte migration before a mature, mechanically competent BM is rebuilt [22, 36]. MMPs are central to this process, coordinating BM breakdown and subsequent repair in response to inflammatory cytokines, growth factors and altered integrin signalling [91, 92]. Recent single‐cell and spatial transcriptomic analyses of acute and chronic human wounds indicate that this programme is highly cell‐type‐specific, with distinct keratinocyte, fibroblast, endothelial and immune subsets expressing characteristic repertoires of MMPs, ADAMs/ADAMTS and serine proteases, thereby compartmentalising protease activity along the wound edge and BM [93, 94, 95, 96].

During wound re‐epithelialisation, keratinocytes switch their integrin repertoire to migrate between the fibrin clot in the wound space and the collagen‐rich dermis, while inducing a broad panel of MMPs [92, 97]. MT1‐MMP mediates pericellular fibrinolysis, activates other MMPs, and basal keratinocyte subpopulations produce MMP‐1, MMP‐3, MMP‐9, MMP‐10, MMP‐19 and MMP‐28 with distinct spatial distributions [98, 99, 100]. In addition to degrading structural BM components, many of these proteases modulate inflammatory and growth‐factor signalling by processing cytokines and chemokines, shedding or activating receptors, and tuning integrin and LM‐332 function at the dermal–epidermal junction, thereby linking BM turnover to keratinocyte migration, angiogenesis and resolution of inflammation [101].

MMP‐9, undetectable in intact epidermis, is induced in migrating keratinocytes, where controlled degradation of collagen IV, LMs and associated receptors permits movement over the wound bed; both deficiency and overexpression of MMP‐9 impair repair, underscoring its dose‐sensitive roles in BM remodelling and subsequent ECM maturation [102, 103, 104, 105]. MMP‐10 further promotes migration by cleaving noncollagenous ECM components, such as LM‐332, while MMP‐19 and MMP‐28 are associated with proliferation and remodelling behind the leading edge [100, 106, 107]. In chronic, nonhealing wounds, persistent inflammatory cues, bacterial biofilms and altered expression of TIMPs and other endogenous inhibitors create a protease imbalance in which sustained MMP and neutrophil‐derived protease activity drives excessive degradation of BM components, growth factors and their receptors, contributing to delayed re‐epithelialisation and repeated BM disruption [108, 109].

**Fig. 2:**
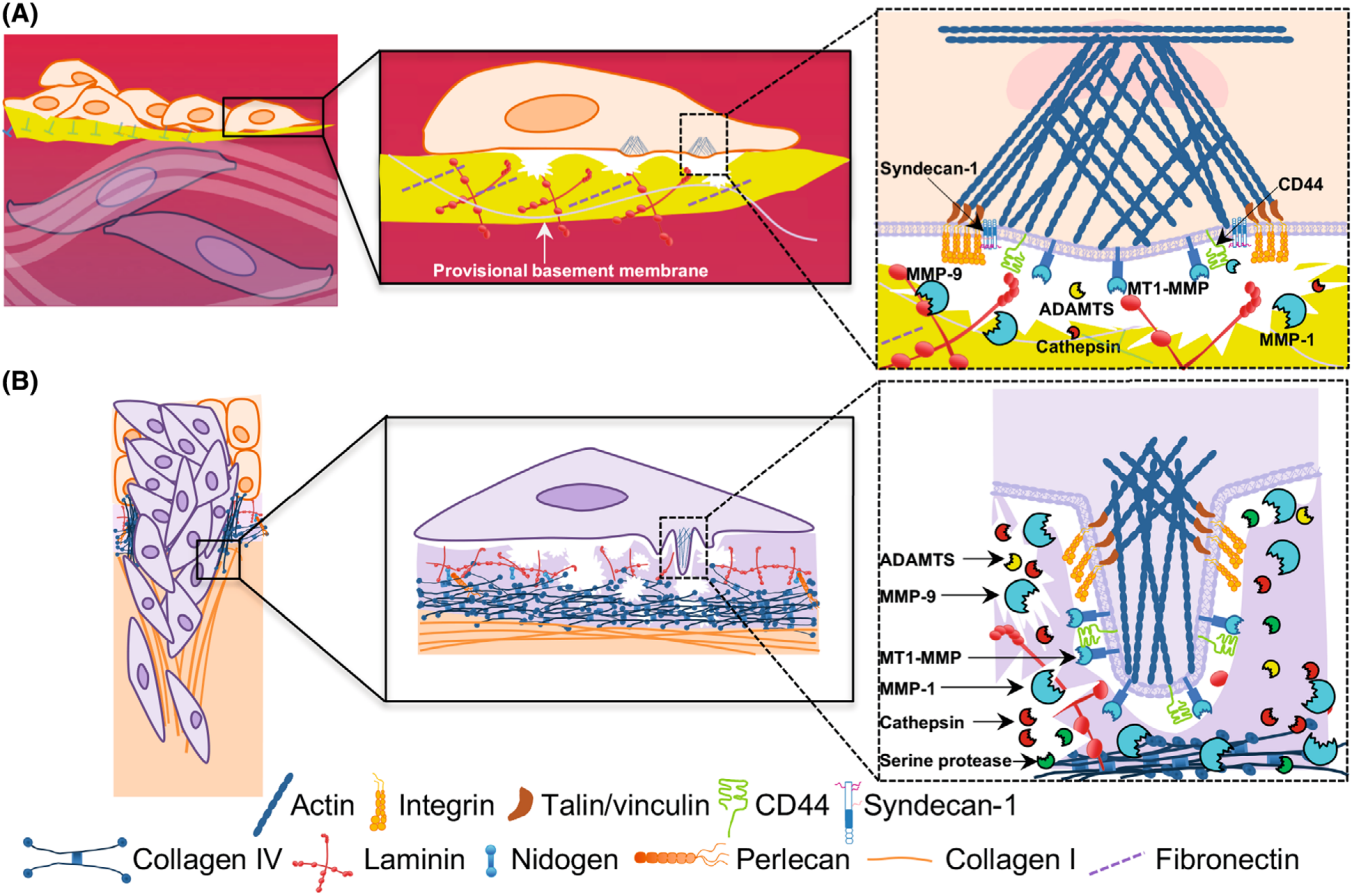
Podosome and invadopodia as context‐dependent basement membrane remodelling machineries in cancer and wound healing. (A) At the edge of a cutaneous wound, leading‐edge keratinocytes assemble podosomes at their basal surface. These invadosome‐like structures contain an F‐actin core surrounded by a ring of integrins and syndecan‐1/CD44 that recruits precursor laminin (LM)‐332 and organises MT1‐MMP/MMP‐9 activity on the basement membrane (BM). Local BM remodelling within podosomes permits directional keratinocyte migration over a provisional matrix, after which a mature dermal–epidermal BM is re‐established. (B) In an invasive carcinoma cell, actin‐rich invadopodia form at the ventral surface, where they concentrate MT1‐MMP together with MMP‐1 and MMP‐9 at discrete sites of contact with the LM and collagen IV‐rich BM. Localised proteolysis generates focal perforations and fragmented collagen IV ‘tracks’ that enable tumour cells to transmigrate across the BM and invade the underlying stroma. Podosome and invadopodia diagram adapted from [111, 198].

Beyond diffuse pericellular proteolysis, migrating keratinocytes concentrate MMP activity within podosomes, actin‐rich adhesion and proteolysis structures previously thought to be restricted to monocytic and transformed cells [110, 111]. These epithelial podosomes form at the basal surface of leading‐edge keratinocytes, where they recruit MT1‐MMP and MMP‐9 and focally remodel LM‐ and collagen IV‐containing BM, thereby facilitating directional migration across the wound bed (Fig. 2A). A key component of this podosome machinery is LM‐332: in intact skin, mature LM‐332 supports stable hemidesmosomal adhesion, whereas during wound healing its precursor form accumulates at the wound edge and acts as a potent motility factor [112, 113, 114, 115, 116]. Precursor LM‐332 reinforces pro‐MMP‐9 expression and cooperates with MT1‐MMP to generate active MMP‐9 within podosomes, where both proteases exert localised proteolytic activity on the underlying matrix [16, 110, 115]. This process depends on syndecan‐1, which, together with CD44, organises a receptor–protease complex around the podosome core to control keratinocyte‐ECM remodelling [16, 110].

As resurfacing completes, dermal–epidermal BM proteins reappear from the wound margins towards the centre in a defined sequence; hemidesmosomes are reconstituted, and basal keratinocytes revert to a stationary, polarised phenotype anchored to the BM [36, 38].

Many of the same MMPs and invadosome components that drive pathological BM breaching in carcinoma, particularly MT1‐MMP and MMP‐9, are transiently mobilised during wound healing to remodel the provisional matrix and BM in a tightly regulated spatiotemporal manner, enabling barrier restoration rather than chronic invasion. In chronic wounds, this spatiotemporal control breaks down, with sustained protease activity and reduced inhibitor levels driving excessive BM degradation and preventing stable reassembly, a pattern that mirrors the pathological BM remodelling observed in cancer invasion. A detailed discussion of translational strategies that exploit this protease dependence, including protease‐modulating and protease‐responsive biomaterials, is provided in the clinical protease inhibition section below [117].

## Invadopodia and protease‐guided basement membrane remodelling in cancer invasion

A central mechanism driving BM remodelling in cancer is the sustained, often mislocalised upregulation of proteases that target BM and peritumoural ECM components, in contrast to the tightly patterned, transient phases of activity that characterise development and acute wound healing. The BM acts as a physical and biochemical barrier that confines carcinoma cells within their tissue of origin; its remodelling and degradation compromise this barrier, enabling local invasion, vascular and lymphatic access and metastatic dissemination, and they serve as important prognostic markers in multiple cancer types [39, 42, 43, 118, 119, 120].

In tumours, most soluble MMPs are produced by fibroblasts, inflammatory cells and endothelial cells within the tumour microenvironment, whereas membrane‐anchored MT‐MMPs are primarily expressed by tumour and stromal cells at invasive fronts [4, 43, 121]. A major consequence of this MMP‐mediated breakdown of pericellular BMs is the creation of permissive paths and growth‐factor‐rich microenvironments that promote tumour cell migration and invasion [1, 122].

Among MT‐MMPs, MT1‐MMP is particularly important for BM breaching in cancer. It localises to actin‐rich invadopodia, where it concentrates proteolytic activity at discrete sites of cell–matrix contact and thereby promotes BM perforation and transmigration (Fig. 2B) [111, 123, 124, 125, 126]. MT1‐MMP directly cleaves key BM constituents, including LMs and collagen IV, and activates pro‐MMP‐2, MMP‐9 and MMP‐13, amplifying pericellular proteolysis [127, 128, 129]. Accordingly, MT1‐MMP expression and activity in cancer cells correlate strongly with metastatic potential and invasiveness [4, 125, 130]. Beyond their static depiction as protease‐rich puncta, invadopodia are now recognised as highly dynamic structures whose assembly, maturation and turnover are tightly coordinated with focal adhesions and regulated by integrins, Src–cortactin–Tks5 signalling, endocytic recycling of MT1‐MMP and local ECM composition [131]. These dynamics enable tumour cells to alternate between phases of matrix degradation and forward translocation, providing the spatiotemporal control necessary for directional invasion [123, 132].

LM and collagen IV networks, as well as cell–ECM adhesion complexes, are targets of MMP‐mediated proteolysis. In vascular BMs enriched in LM‐411 and LM‐511, such remodelling facilitates extravasation by enabling tumour cells to cross the endothelial BM [133]. Although many MMPs can degrade BM proteins, invasive tumour cells rely predominantly on MMP‐2, MMP‐9 and MT1‐MMP, often concentrated in invadopodia [134, 135, 136, 137, 138, 139, 140, 141, 142]. Increased collagen IV degradation in human tumours, mainly MT‐MMP‐dependent, correlates with enhanced metastatic potential, underscoring the functional importance of collagen IV as a barrier to dissemination [32, 118, 119, 143]. In several tumour types, type IV collagen does not simply disappear but undergoes irregular degradation and linear redeposition, forming discontinuous sheaths and ‘escape tracks’ that guide collective invasion and intravasation [4, 144].

The consequences of LM degradation in cancer are complex, as proteolysis can both eliminate adhesive cues and generate bioactive fragments with novel signalling activities. Destruction of LM‐111 by MMP‐9 may disrupt homeostatic signals that restrain epithelial proliferation at early stages of breast cancer, whereas elastase/MMP‐9‐mediated remodelling of LM‐111 can expose cryptic epitopes that awaken dormant breast cancer cells via α3β1 integrin signalling, with LM‐211, LM‐411 and LM‐511 likely affected in a similar manner [145, 146, 147]. LM α1, β1 and γ1 chains are substrates for MMP‐2, MMP‐9 and MT1‐MMP, yielding fragments that signal through integrins and syndecans and can promote inflammatory, pro‐migratory and pro‐angiogenic responses, including in Ras‐induced EMT models and prostate cancer cells [148, 149, 150, 151, 152, 153, 154].

LM‐332 has attracted particular attention in cancer because of the many proteolytic cleavages affecting its three subunits and its dual role as a stable anchoring ligand and a pro‐migratory substrate [116, 155, 156]. Under physiological conditions, LM‐332 undergoes tightly regulated maturation processings that enable its supramolecular integration into epithelial BMs and its anchoring function through hemidesmosomal complexes [116, 157, 158, 159]. In cancers, additional cleavage events alter both its adhesive and linking functions and simultaneously generate fragments that promote motility, invasion and survival [155]. For instance, cleavage of the β3 chain by MMP‐7 or MT1‐MMP enhances colon and prostate carcinoma cell migration and invasion, respectively, whereas MMP‐2 and MT1‐MMP cleavage of the γ2 chain generates N‐terminal LE fragments that trigger EGF receptor signalling and downstream MAPK activation in tumour cells [160, 161, 162, 163, 164, 165]. Additional MMPs (MMP‐3, MMP‐12, MMP‐13, MMP‐19 and MMP‐20) can also process γ2, reinforcing the status of LM‐332, and especially its γ2 subunit, as a prime target for MMPs in tumourigenesis [139, 140, 146, 166].

While the *in vivo* contribution of soluble collagenases such as MMP‐2 and MMP‐9 to degradation of the highly cross‐linked collagen IV network remains debated, there is strong consensus that MT‐MMPs are crucial for collagen IV dissolution and BM transmigration by cancer cells [32, 44]. MT‐MMPs such as MT1‐MMP remodel BMs not only by cleaving LMs and collagen IV but also by enlarging and coalescing pre‐existing pores, converting a continuous collagen IV network into a permissive, fenestrated barrier for invasive cells [130, 143, 167, 168]. In addition to LMs and collagen IV, linker and reservoir BM components, such as nidogen, perlecan and agrin are also susceptible to proteolysis, which can destabilise LM‐collagen IV cross‐linking and alter growth factor presentation within tumour BMs [39, 169].

**Fig. 3:**
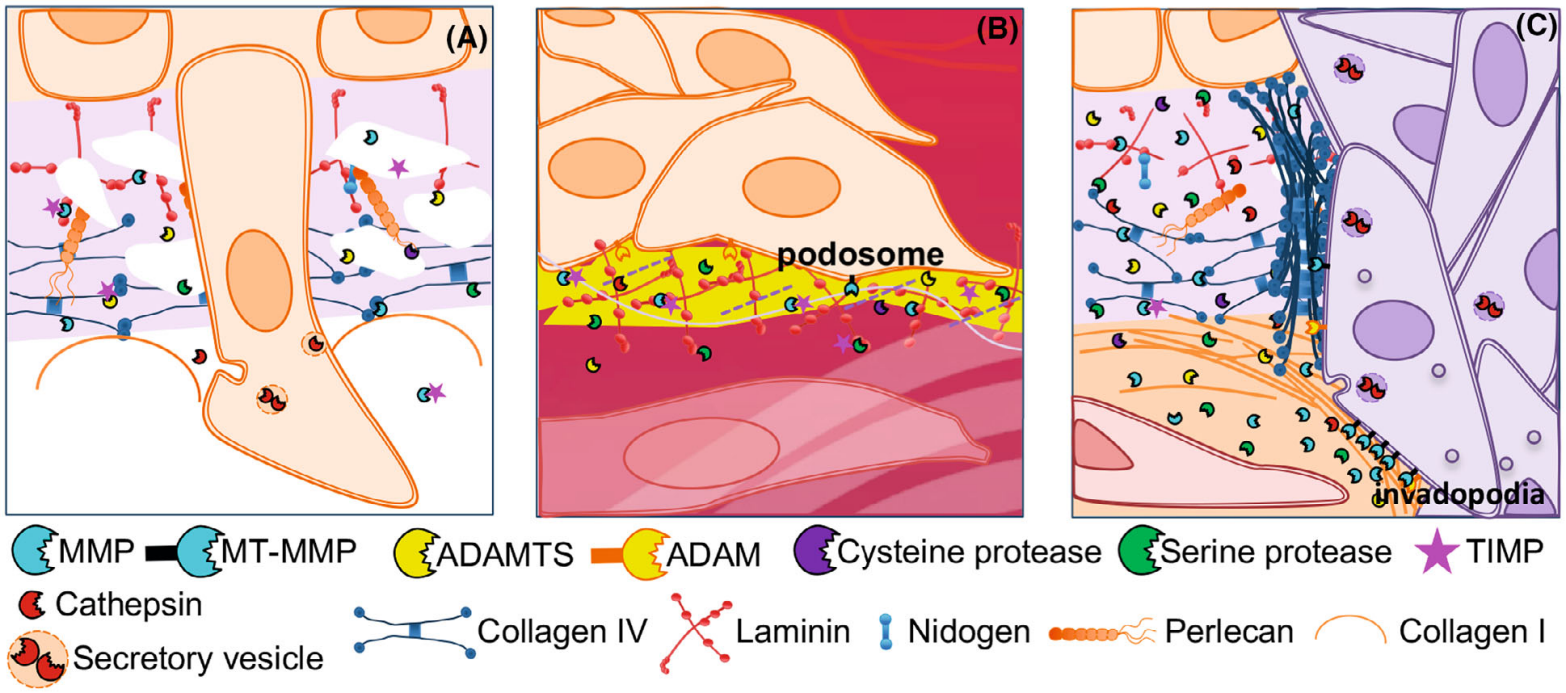
Shared protease toolkit, distinct levels and spatiotemporal control of basement membrane remodelling in development, wound healing and cancer. Schematic overview of basement membrane (BM) remodelling across three contexts that reuse a common protease toolkit: MMPs, ADAMs/ADAMTS, serine proteases and cysteine proteases such as cathepsins, but differ in total protease load, spatial distribution and timing, leading to distinct outcomes. (A) In development, low‐to‐moderate, tightly patterned and transient phases of MMP, ADAMTS and selected serine/cathepsin activities are confined to specific cell‐BM interfaces (e.g. branching tips or tissue boundaries), permitting focal BM perforation, sliding and reassembly while overall barrier integrity is preserved. (B) In acute wound healing, there is a transient local increase in protease activity at the wound edge: MMPs, ADAMs/ADAMTS, serine proteases (e.g. neutrophil elastase and plasmin) and cathepsins contribute to BM degradation and provisional matrix turnover, but their expression is spatially restricted and rapidly counterbalanced by TIMPs and other inhibitors, allowing re‐establishment of a mature, mechanically competent BM. (C) In cancer invasion, the same protease families are upregulated to higher and more persistent levels and become mislocalised (e.g. MT1‐MMP concentrated at invadopodia or basolateral membranes, sustained MMP‐2/MMP‐9, elevated elastase, plasmin and cathepsins throughout the wound bed or tumour–stroma interface), while inhibitor networks are perturbed. This combination of increased protease burden, loss of spatial restriction and impaired inhibition drives chronic BM weakening, formation of discontinuous collagen IV ‘escape tracks’ and invasive paths, and failure to restore barrier integrity, illustrating how differences in both protease amount and spatiotemporal regulation, rather than protease identity alone, distinguish physiological from pathological BM *remodelling*.

Invadopodia also contribute to collective invasion, where specialised leader cells at the invasive front form protease‐rich protrusions that carve paths through the BM, including collagen IV ‘escape tracks’, and into the surrounding stromal ECM, along which follower cells migrate. In such strands, leader cells show elevated invadopodia activity and MMP/MT1‐MMP localisation compared with followers, linking invadopodia dynamics to the organisation of invasive fronts and metastatic potential (Fig. 3C) [123, 170].

Invading cells can also apply mechanical forces that promote physical rupture of BM architecture [39, 171], but massive protease production by cancer cells greatly enhances their invasive capacity. Emerging work further shows that the mechanical properties of the tumour microenvironment strongly influence invadopodia formation and function. Increased matrix stiffness can enhance invadopodia number and degradative activity and promote a more mesenchymal, invasive morphology in several cancer types, via pathways involving β1‐integrin, cortactin and PI3K/AKT signalling. In three‐dimensional BM‐like matrices, stiffness and confinement also modulate invadopodia length, lifetime and the balance between degradative and purely mechanical protrusive activity, underscoring that invadopodia respond to both biochemical and physical cues *in vivo* [172].

Nonproteolytic mechanisms can also play a decisive role in metastasis: *in vitro*, tumour cells can switch to protease‐independent migration when protease activity is blocked, indicating that proteases are not always strictly required for invasion [173, 174]. In line with this, while cancer cells often breach BM barriers via protease‐dependent mechanisms involving invadopodia‐associated MT‐MMPs, immune cells such as macrophages can instead exploit pre‐existing BM pores in a largely protease‐independent, actomyosin‐driven manner [40].

## Cathepsins and additional protease systems in basement membrane remodelling

Cysteine cathepsins also participate in BM and pericellular matrix remodelling and have emerged as important extracellular proteases alongside MMPs and ADAMTS [175]. Previously regarded as primarily lysosomal hydrolases, cathepsins B, K, L and S are now known to be secreted or displayed at the cell surface, where they can function at mildly acidic or near‐neutral pH to cleave ECM components. *In vitro*, cathepsins B and L degrade native BM‐like matrices and major ECM constituents, including collagen and LMs, and can release bioactive fragments and latent protease activities from matrix digests, indicating both direct and indirect contributions to BM and ECM dissolution [176]. At the dermal–epidermal junction, cathepsin S cleaves nidogen‐1 and nidogen‐2 within their C‐terminal G2/G3 domains, impairing their ability to bridge LM and collagen IV networks and suggesting that cathepsin‐mediated processing can destabilise BM architecture and alter its mechanical properties [177]. In cancer and vascular disease, elevated cathepsin B, L and S activities correlate with increased invasion, angiogenesis and inflammatory remodelling, where secreted cathepsins cooperate with MMPs to remodel BM‐adjacent ECM and modulate growth factor and cytokine availability [178]. Recent reports emphasise that extracellular cathepsins not only degrade ECM/BM but also shed receptors and process chemokines, thereby linking proteolysis to cell migration, immune cell recruitment and chronic inflammation [179]. Conversely, in wound and tissue repair, dysregulated cathepsin activity can impair healing through excessive matrix and BM degradation, or, when appropriately controlled (e.g. cathepsin K inhibition in diabetic wounds), contribute to more effective remodelling and regeneration [180].

Beyond MMPs, ADAMs/ADAMTS and cathepsins, only a few additional protease classes have reasonably direct links to BM‐adjacent remodelling: serine proteases (plasmin, elastase and uPA‐dependent cascades) and BM‐associated regulators such as papilin can modulate LM and collagen IV turnover, often by activating or cooperating with MMPs and cathepsins [53, 178].

## Clinical protease inhibition/modulation at the basement membrane

Attempts to therapeutically modulate BM remodelling have primarily focused on inhibiting MMPs, given their well‐established roles in BM degradation during cancer invasion, fibrosis and inflammatory disease. First‐generation broad‐spectrum small‐molecule MMP inhibitors (e.g. batimastat, marimastat, prinomastat and tanomastat) were tested in multiple Phase II–III trials for solid tumours but generally failed to improve survival and frequently caused dose‐limiting musculoskeletal pain and inflammation. These outcomes are now thought to reflect both inadequate target selectivity and disruption of physiological tissue remodelling (Table 2) [181, 182]. This experience led to the development of more selective approaches, including isoform‐restricted small molecules, tetracycline‐derived broad inhibitors, such as doxycycline and incyclinide, and monoclonal antibodies against MMP‐2/MMP‐9 and MT1‐MMP, which have shown acceptable safety and target engagement but so far limited efficacy signals in oncology and cardiovascular indications (Table 2) [183]. Recent efforts have produced increasingly selective, nonhydroxamate small molecules, noncatalytic‐site protease inhibitors and antibody‐based MMP blockers, but these next‐generation agents remain largely in preclinical or very early‐phase evaluation, and no new MMP inhibitor has yet demonstrated clear, reproducible clinical benefit in BM‐associated diseases, such as invasive cancer or chronic wounds [184].

**Table 2:** Selected clinical trials of matrix metalloproteinase inhibitors, including tetracycline‐derived modulators.

Inhibitor (type)	Main MMP targets	Cancer type/setting (or other indication)	Trial phase and status	Key outcome
Batimastat (BB‐94), marimastat (BB‐2516); small‐molecule hydroxamates	Broad MMPs (MMP‐1, MMP‐2, MMP‐3, MMP‐7, MMP‐9, etc.)	Pancreatic, lung, breast and others	Multiple Phase II–III, completed	No survival benefit; significant musculoskeletal toxicity: development halted [183, 195]
Prinomastat (AG3340), tanomastat (BAY 12‐9566)	Broad MMPs, designed to spare some MMPs	Nonsmall‐cell lung cancer, pancreatic cancer	Phase III, completed	No improvement in overall survival; some trials showed worse outcomes vs control [182, 183, 195]
Andecaliximab (GS‐5745); monoclonal antibody	MMP‐9	Advanced gastric/gastro‐oesophageal junction cancer (with chemotherapy)	Phase II/III, completed	Early‐phase II suggested activity, but Phase III failed to show overall or progression‐free survival benefit [183, 196]
Experimental anti‐MT1‐MMP antibodies/inhibitors	MT1‐MMP	Solid tumours (early trials/preclinical)	Preclinical/early phase	Inhibition of invasion and angiogenesis in models; no positive late‐phase clinical data yet [185]
Doxycycline (subantimicrobial‐dose tetracycline)	Broad, relatively weak inhibitor, preferential for MMP‐8, MMP‐9 and other collagenases/gelatinases	Cardiovascular disease (e.g. abdominal aortic aneurysm, atherosclerotic plaque), chronic inflammatory states	Mainly phase II, completed	Reduced MMP‐1, MMP‐8, MMP‐9 expression and activity in vascular lesions and inflammatory settings; some biomarker and imaging improvements, but no consistent demonstration of hard clinical‐outcome benefit [197]
Incyclinide (CMT‐3/COL‐3); chemically modified tetracycline	Broad MMP inhibitor (MMP‐2, MMP‐9 and others) with reduced antimicrobial activity	Kaposi's sarcoma, other solid tumours; dermatologic indications (acne, rosacea)	Phase II oncology/dermatology trials, completed/halted	Well tolerated; in some studies, reduced circulating MMP‐2/MMP‐9 with antitumour or anti‐inflammatory activity, but larger trials in several indications failed to show clear efficacy: development curtailed

Beyond MMPs, other protease families that contribute to BM‐adjacent ECM remodelling have also been targeted clinically or preclinically. ADAM and ADAMTS metalloproteinases are under investigation in inflammatory disease, arthritis and cancer, but selective inhibitors are only beginning to enter early‐phase trials and no agent has yet demonstrated clear benefit in BM‐centred pathologies [54, 185]. Cysteine cathepsin inhibitors, particularly against cathepsin K and S, have reached Phase II–III in osteoporosis and autoimmune diseases, providing proof‐of‐concept that these enzymes are druggable, although several programmes, such as the cathepsin K inhibitor odanacatib, were halted despite robust fracture‐risk reduction because of on‐target adverse events, underscoring the risks of systemic inhibition of broadly expressed ECM proteases [186]. Serine protease inhibitors are already approved or in trials for other indications, including coagulation disorders, pancreatitis, viral entry and acute lung injury, but their potential to selectively modulate BM remodelling in chronic wounds or cancer remains largely unexplored [93, 187, 188].

In addition to systemic small‐molecule and antibody‐based inhibitors, local protease‐modulating biomaterials have emerged as a complementary strategy to rebalance protease activity at the wound‐BM interface [93, 117]. Protease‐modulating dressings, such as polyacrylate‐based hydrogels and other superabsorbent or lipido‐colloid dressings, can bind excess MMPs and divalent cofactors in chronic wound fluid and have been reported to normalise protease levels and improve granulation and closure in venous leg ulcers and other hard‐to‐heal wounds [189, 190]. More recently, protease‐responsive hydrogels and peptide‐based scaffolds, cross‐linked with protease‐cleavable linkers or bioactive components, such as curcumin‐derived carbon dots, have been designed to sense elevated protease activity and, in response, release growth factors, antimicrobials or matrix components in proportion to local protease levels, accelerating re‐epithelialisation and BM reassembly in preclinical models [191, 192]. In parallel, immunomodulatory biomaterials that combine protease modulation with controlled presentation of cytokines, chemokines or matricellular proteins aim to dampen chronic inflammation and restore a more regenerative protease‐inhibitor balance at the dermal–epidermal junction in nonhealing wounds, offering a more nuanced alternative to systemic, active‐site‐directed protease inhibition [193, 194].

## Physiological versus pathological basement membrane remodelling: conclusions and future perspectives

Throughout development, wound healing and cancer, BM remodelling relies on a shared protease toolkit but differs fundamentally in its duration, spatial patterning and outcome (Fig. 3). During morphogenesis and acute cutaneous repair, MMPs, ADAM/ADAMTS and other proteases are expressed in a tightly patterned, cell‐type‐specific manner, activated briefly at defined interfaces such as branching tips or the wound edge, and rapidly rebalanced by local inhibitors. This enables transient BM perforation and reassembly, supporting cell migration and tissue reshaping without long‐term barrier loss (Fig. 3A,B). In contrast, tumour invasion and chronic wounds are characterised by persistent or repeatedly reactivated protease expression, mislocalisation of key enzymes such as MT1‐MMP to basolateral membranes and invadopodia, and disruption of the local protease‐inhibitor balance, resulting in chronic BM weakening, invasive track formation and progressive tissue destabilisation (Fig. 3C). A key insight from these comparisons is that the distinction between physiological and pathological BM remodelling lies less in the identity of the proteases involved than in their spatiotemporal control and in the integrity of the surrounding regulatory networks, particularly the balance between MMPs, ADAM/ADAMTS proteases and their inhibitors at the BM.

Looking ahead, an important challenge will be to integrate biochemical, mechanical and spatial information into quantitative *in vivo* models of BM remodelling. Combining multiplexed imaging and single‐cell or spatial omics with fluorophore‐tagged BM components, biosensors for protease activity and computational approaches to BM dynamics should clarify how protease networks, inhibitors and BM mechanics interact in real time during development, wound healing and tumour invasion. Translationally, next‐generation strategies, including isoform‐selective or noncatalytic‐site protease inhibitors, protease‐responsive biomaterials and engineered BM‐mimetic hydrogels that independently tune BM ligand composition and stiffness, offer promising avenues to locally modulate protease activity and BM mechanics in chronic wounds and cancers, but they will require careful validation to ensure restoration, rather than further compromise, of BM integrity. In this context, our work on LM‐332 reorganisation and MT1‐MMP/MMP‐9‐dependent podosome activity at the dermal–epidermal junction provides a mechanistic framework to understand how epithelial cells locally tune BM integrity during both efficient wound repair and early stages of malignant invasion.

## Conclusion

Taken together, these observations suggest that BM remodelling should be seen less as a unidirectional degradation process and more as a tightly regulated, context‐specific programme integrating proteases, inhibitors, and mechanics. Dissecting and selectively re‐tuning this programme *in vivo* offers an opportunity to restore barrier function in chronic wounds and to restrain invasive growth in cancer without disrupting the physiological BM dynamics required for development and tissue homeostasis.

## Author contributions

PR wrote the manuscript. CL made figures. PR, CL and GD edited the manuscript.

## Conflict of interest

The authors declare no conflict of interest.
